# T228. VARIABILITY AND UNDERUTILISATION OF CLOZAPINE IN SPAIN

**DOI:** 10.1093/schbul/sby016.504

**Published:** 2018-04-01

**Authors:** Francisco Javier Sanz-Fuentenebro, Jose Juan Uriarte Uriarte, Pere Bonet Dalmau, Vicente Molina Rodriguez, Alba Lubeiro Juárez, Miguel Bernardo Arroyo

**Affiliations:** 1 Hospital 12 de Octubre; 2 Gestión Clínica, Red de Salud Mental de Bizkaia; 3 Salud Mental Fundación Althaia, “Consell Assessor de Salut Mental”; 4 University of Valladolid; 5 Hospital Clínic, Universitat De Barcelona, IDIBAPS, CIBERSAM

## Abstract

**Background:**

The analysis of the information available in many countries on the use of clozapine, systematically indicates low prescription, underdosing and delay in the start of treatment. But as striking as this underutilization is the great variability between territories studied, which are related to multiple factors, and have led to various initiatives to improve its use. We do not have studies that evaluate these aspects in the Spanish population, so we have considered a first approximation through samples from four territories.

**Methods:**

The authors analyzed the prescription data of clozapine in Castilla y León, the Basque Country, Catalonia and a Southern Madrid Area.

**Results:**

The patients diagnosed with schizophrenia who receive treatment in the territories studied oscillate around 0.3%; the treatments with clozapine / 10000 inhabitants between 33.0 and 57.0; and patients diagnosed as schizophrenia receiving clozapine account for between 13.7% and 17.9% of those treated. The coefficient of variation between centers and prescribers is frequently higher than 50%.

**Discussion:**

The global clozapine prescription data in the territories studied are in the range of countries in our environment. The variability in the prescription is very high and increases as we analyze smaller territories, until a great heterogeneity of the individual prescription.

